# BiodivNERE: Gold standard corpora for named entity recognition and relation extraction in the biodiversity domain

**DOI:** 10.3897/BDJ.10.e89481

**Published:** 2022-10-07

**Authors:** Nora Abdelmageed, Felicitas Löffler, Leila Feddoul, Alsayed Algergawy, Sheeba Samuel, Jitendra Gaikwad, Anahita Kazem, Birgitta König-Ries

**Affiliations:** 1 Heinz Nixdorf Chair for Distributed Information Systems, Department of Mathematics and Computer Science, Friedrich Schiller University Jena, Jena, Germany Heinz Nixdorf Chair for Distributed Information Systems, Department of Mathematics and Computer Science, Friedrich Schiller University Jena Jena Germany; 2 Michael-Stifel-Center for Data-Driven and Simulation Science, Jena, Germany Michael-Stifel-Center for Data-Driven and Simulation Science Jena Germany; 3 German Center for Integrative Biodiversity Research (iDiv), Halle-Jena-Leipzig, Germany German Center for Integrative Biodiversity Research (iDiv) Halle-Jena-Leipzig Germany

**Keywords:** entity annotation, relation annotation, Named Entity Recognition (NER), Relation Extraction (RE), Information Extraction (IE), biodiversity research, gold standard

## Abstract

**Background:**

Biodiversity is the assortment of life on earth covering evolutionary, ecological, biological, and social forms. To preserve life in all its variety and richness, it is imperative to monitor the current state of biodiversity and its change over time and to understand the forces driving it. This need has resulted in numerous works being published in this field. With this, a large amount of textual data (publications) and metadata (e.g. dataset description) has been generated. To support the management and analysis of these data, two techniques from computer science are of interest, namely Named Entity Recognition (NER) and Relation Extraction (RE). While the former enables better content discovery and understanding, the latter fosters the analysis by detecting connections between entities and, thus, allows us to draw conclusions and answer relevant domain-specific questions. To automatically predict entities and their relations, machine/deep learning techniques could be used. The training and evaluation of those techniques require labelled corpora.

**New information:**

In this paper, we present two gold-standard corpora for Named Entity Recognition (NER) and Relation Extraction (RE) generated from biodiversity datasets metadata and abstracts that can be used as evaluation benchmarks for the development of new computer-supported tools that require machine learning or deep learning techniques. These corpora are manually labelled and verified by biodiversity experts. In addition, we explain the detailed steps of constructing these datasets. Moreover, we demonstrate the underlying ontology for the classes and relations used to annotate such corpora.

## Introduction

The increasing amount of scientific datasets in public data repositories calls for more intelligent systems that automatically analyse, process, integrate, connect or visualise data. An essential building block in the evolution of such computer-supported analysis tools is Information Extraction with its sub-tasks, Named Entity Recognition (NER) and Relation Extraction (RE). That process aims to automatically identify important terms (entities) and groups of terms/expressions, which can fall into a certain category in the data (NER), as well as relationships between these entities (RE). However, the advancement of such tools is applicable if *gold standards*, manually labelled test corpora, are available. This supports the training of machines (for machine-learning approaches) and allows an evaluation of the developed tool. For applied domains, such as biodiversity research, gold standards are very rare.

In this work, we present a novel gold standard for biodiversity research. We provide a NER corpus, based on scientific metadata files and abstracts with manual annotations of important terms, such as species (ORGANISM), environmental terms (ENVIRONMENT), data parameters and variables measured (QUALITY), geographic locations (LOCATION), biological, chemical and physical processes (PHENOMENA) and materials (MATTER), for example, chemical compounds. In addition, we provide an RE corpus, based on a portion of the same data that consists of important binary and multi-class relationships amongst entities, such as OCCUR_IN (Organism, Environment), INFLUENCE (Organism, Process) and HAVE/OF (Quality, Environment). We also added these identified entities and relationships to a conceptual model developed in our previous work (Abdelmageed et al. 2021a).

Our contribution is threefold:

- a NER corpus, based on metadata and abstracts with the following entities: ORGANISM, ENVIRONMENT, QUALITY, LOCATION, PHENOMENA, MATTER
- an RE corpus, based on a portion of the same data, with the following relationships containing the entities identified in the NER corpus: OCCUR_IN, INFLUENCE and OF/HAVE
- a conceptual model that integrates all concepts and relations.

We provide the results in formats that allow easy further processing for various Natural Language Processing (NLP) tasks, based on machine-learning and deep learning techniques. The code and the data are publicly available as follows:

1. The data DOI: https://doi.org/10.5281/zenodo.6575865
2. Github Repo for the scripts: https://github.com/fusion-jena/BiodivNERE

### Background

Biodiversity research is a sub-research domain of the Life Sciences that comprehends the totality and variability of organisms, their morphology and genetics, life history and habitats and geographical ranges (Shanmughavel 2007). Scientific data generated in biodiversity research are very heterogenous and can occur in multiple formats. This is an obstacle for machine processing, which needs additional information for data integration, data search or data visualisation. Therefore, primary research data are described by metadata and descriptive information along the W-questions (what, who, when, where and why). Such metadata are mostly provided in structured formats, such as JSON or XML.

Natural Language Processing (NLP), with its sub-task Information Extraction, is a research field that uses these structured data or scientific publications. The aim is to develop systems that automatically identify important terms and phrases in the text. That supports scholars in obtaining a quick overview of unknown texts, for example, in search or allows improved filtering. In the Life Sciences, Information Extraction has a long history (Thessen et al. 2012). Driven by a series of workshops and shared tasks, such as BioNLP, BioCreative and BioASQ in the scope of CLEF, multiple corpora and tools for various purposes were developed to extract main entities from text and relations amongst them automatically. However, determining what a relevant entity or relation in a document or data depends on the domain of focus. While scholars looking for biomedical data are mainly interested in data types, such as diseases, biological processes and organisms (Roberts et al. 2017) and related entities, such as genes and proteins, in biodiversity research, other categories are of relevance, namely: organisms, environmental terms, geographic locations, measured data parameters, materials, biological, physical and chemical processes and data types (Löffler et al. 2021).

### Previous Resources Analysis

In the first step, we had to figure out which categories (or entity types) are relevant for biodiversity research. In addition, we also had to explore occurring relations amongst these entities. Therefore, we selected two sources from our previous works: 1) BiodivOnto (Abdelmageed et al. 2021b) and a biodiversity research-related question corpus (Löffler et al. 2021). In this section, we describe how we decided on the classes and relations to be used in the annotation process. We also elaborate on how we came along with a reconciled model representing the final conceptual model we used in this work. In addition, we introduce the underlying data sources for the development of the novel gold standards.

**Biodiversity Questions**

The biodiversity question corpus consists of 169 questions provided by around 70 scholars of three biodiversity research-related projects (Löffler et al. 2021). Concerning the topics and granularity, the questions are very diverse and reflect different information needs. While some questions ask for facts, such as "What butterfly species occur on calcareous grassland?", others are more complex and aim to obtain an answer on associations, for example, How do autotrophic microorganisms influence carbon cycling in groundwater aquifers? The noun entities of these questions were manually labelled (including nested entities, such as adjectives, for example, autotrophic microorganisms). Nine biodiversity scholars grouped the labelled nouns and phrases into 13 proposed categories. Each annotator classified all 169 questions, which resulted in 592 total annotations. It turned out that seven categories (entity types) were mentioned very often (at least 89 times per category): ORGANISM (e.g. plants, fungi, bacteria), ENVIRONMENT (environments species live in), QUALITY (characteristics to be measured), MATERIAL (e.g. chemical compounds), PROCESS (re-occurring biological and physical processes), LOCATION (geographic location) and DATA TYPE (research results, e.g. lidar data). All annotations for which the inter-rater agreement was larger than 0.6 (representing a substantial agreement (Landis and Koch 1977)) were exported to a final XML file.

The identified relevant entity types from this question corpus were aligned with the detected categories of classes from BiodivOnto in several discussion rounds. The final outcome (see Table 1) was used to inspect the annotated questions again. This inspection consists of manually detecting the relations between the already annotated entities in each question. We omitted questions that do not possess any annotation of the final classes or provide only one class. We only considered questions that contain at least two annotations of the entity types in Table 1. In total, 91 questions were utilised for the relation detection in the question corpus.

The main idea for the relation detection process was to come up with categorisation for relations similar to the categories for noun entities. The detection process was conducted in several rounds. In the first pilot phase, three scholars analysed only a few questions about the existence of relations. The initial instruction was to manually inspect the questions and to identify binary relations between the occurring entities. Scholars were also advised to inspect the given verbs (which mainly describe a relation) and to think about suitable categories for the relations. In a second round, the proposed relation categories were discussed. The outcome was used for the final detection round. The final agreed relation categories are:

- influence (an entity influences another entity, for example, an ORGANISM influence PHENOMENA),
- occur (an entity occurs in another entity, for example, PROCESS occur ENVIRONMENT),
- of (inverse relation of have: an entity of an entity or an entity has another entity, for example, QUALITY of ORGANISM)

**Figure 1.**
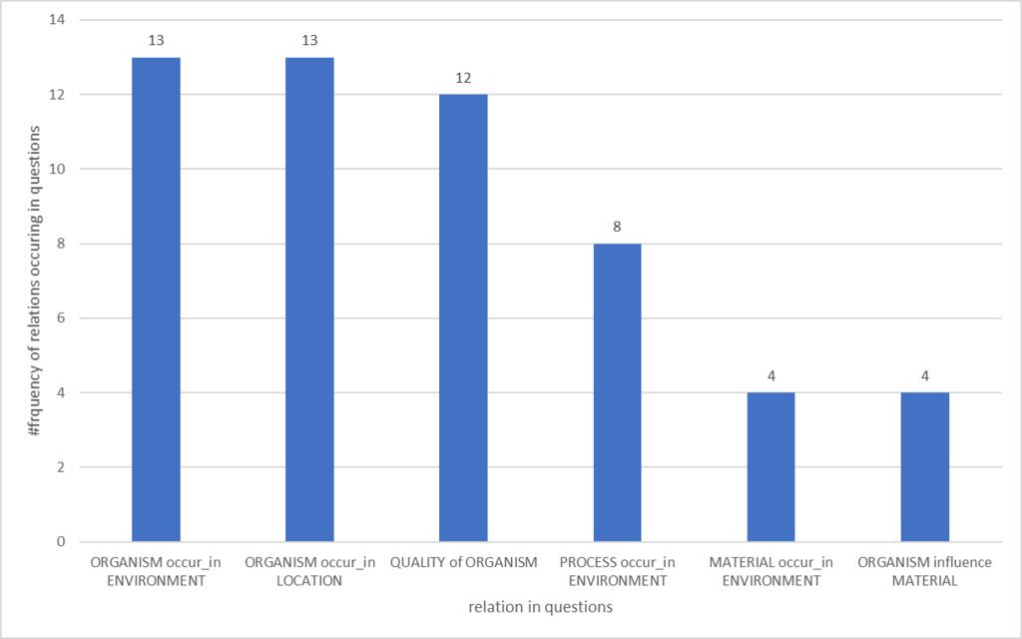
Occurrence frequency of relations in questions related to biodiversity research.

Complex questions with several entities were split into several relations. For example, the question "How do (autotrophic microorganisms)[ORGANISM] influence (carbon cycling) (PHENOMENA) in (groundwater aquifers)[ENVIRONMENT]"? This resulted in detecting two relations: influence (autotrophic microorganisms _ORGANISM_, carbon cycling _PHENOMENA_) and occur (carbon cycling _PHENOMENA_, groundwater aquifers _ENVIRONMENT_). Fig. 1 presents the outcome of the relation detection of the question corpus. The most frequent relation patterns are ORGANISM *occur* ENVIRONMENT and ORGANISM *occur* LOCATION, with 13 mentions each. This result served as input for the conceptual model, as well as for the subsequent relation annotation of metadata and abstracts.

**BiodivOnto**

BiodivOnto is a conceptual model of the core concepts and relations in the biodiversity domain. The first version of BiodivOnto (Abdelmageed et al. 2021b) was developed in 2021, whereas the most recent ontology version is given by (Abdelmageed et al. 2021a). Such core or general concepts represent the classes of annotation utilised. The proposed class names were discussed with two biodiversity experts who are also authors of this paper. We finally agreed on, for example, ORGANISM, PHENOMENA and MATTER as tags for the NER corpus. However, BiodivOnto contains subclasses as well, like Ecosystem and Landscape, which are subclasses of the Environment class. To facilitate the annotation process, we decided to use the top-level classes only. In this case, both Ecosystem and Landscape are substituted by the ENVIRONMENT class. The same applies to Trait and Quality, where only QUALITY was used as an annotating class. LOCATION has appeared as a common concept in the Biodiversity Questions (see above); we included it as well as a core concept in the BiodivOnto. Table 1 summarises the final selected classes of interest that were used in the NER annotation.

BiodivOnto initially had the following relations:

- have: that appeared between ORGANISM-ENVIRONMENT, ORGANISM-QUALITY, ENVIRONMENT-QUALITY and MATTER-QUALITY.
- occur_in: that appeared between PHENOMENA-ENVIRONMENT.

However, we merged the outcome from the analysis of the Biodiversity Questions as we did for classes. Thus, we included new relations as follows:

- occur_in linking MATTER-ENVIRONMENT, ORGANISM-LOCATION, ORGANISM-ORGANISM, PHENOMENA-LOCATION and ENVIRONMENT-LOCATION.
- influence relating ORGANISM-PHENOMENA, ORGANISM-MATTER, PHENOMENA-PHENOMENA, PHENOMENA-QUALITY, PHENOMENA-ENVIRONMENT and QUALITY-QUALITY.

On the other hand, BiodivOnto initially included both "part_of" and "is_a" relations. However, we do not include them in the new ontology version since the most common relations in the Biodiversity Questions lack them. We picked on the relations that appear in both sources only.

**Figure 2.**
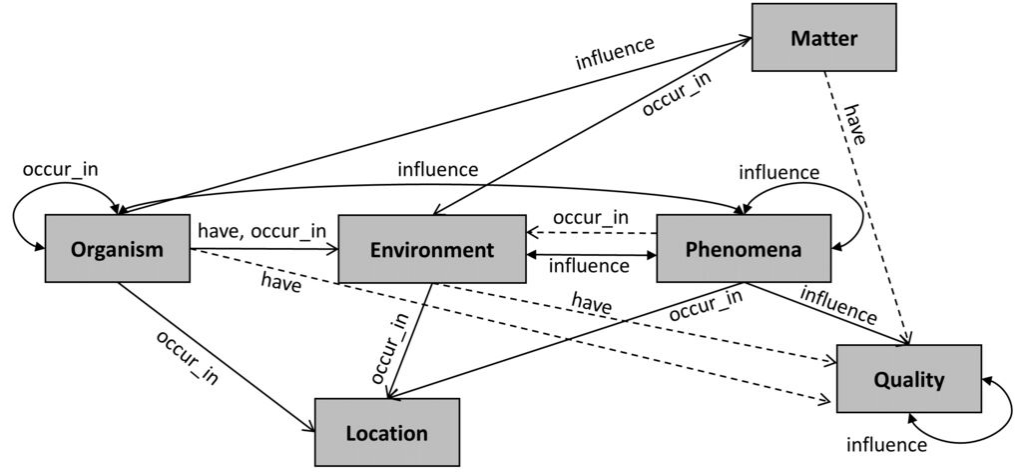
Updated version of BiodivOnto. Dashed lines are relations from the original BiodivOnto, while solid lines are the new ones, based on the Biodiversity questions.

Fig. 2 illustrates the reconciled version of BiodivOnto, based on the old BiodivOnto model and the Biodiversity Questions. It consists of six classes and 17 relations we used in the annotation process.

**Data Sources**

To construct our corpora, we re-used our previous work's collected metadata and abstracts (Abdelmageed et al. 2021b). Thus, metadata files are gathered from two data sources with very different characteristics (BEFChina and data.world). The Semedico search engine (Faessler and Hahn 2017) retrieves relevant abstracts from PubMed, a source with more than 32M abstracts. To ensure the relevance of the crawled data from Semedico, we have followed an iterative way of revision. We started with the initial keywords set that we used to crawl. Then, we manually revised it to guarantee relevance. More details on the collection and crawling, license verification, and biodiversity relevance checking are already explained in (Abdelmageed et al. 2021b) and go beyond the scope of this paper. Initially, these collected data were meant to extract biodiversity-related keywords. However, in this work, we use them for the purpose of developing NER and RE corpora.

### Related Work

The loss of biodiversity has a lot of concerns and it considers a major issue in our life (Butchart et al. 2010, Cardinale et al. 2012). Research in this domain has recently seen accelerated growth, leading to the big data scenario of the biodiversity literature. For instance, the Biodiversity Heritage Library (BHL) currently holds over 55 million digitised pages of legacy biology text from the 15^th^-21^st^ centuries, representing a huge amount of textual content (Nguyen et al. 2019). Extracting core knowledge, i.e. entities and relations between these entities, from myriads of available resources, allows a better overview of the data and thus supports fact discovery. In this section, we outline the state-of-the-art related work towards building such gold standards in the Life Sciences, focusing on biodiversity research.

**Named Entity Recognition (NER) Corpora**

BIOfid (Ahmed et al. 2019) is a Specialised Information Service for Biodiversity Research launched to mobilise valuable biological data from printed literature hidden in German libraries for the past 250 years. First, historical literature was converted into text using OCR and plants, birds and butterfly occurrences were annotated. A training dataset was then generated for named entity recognition and taxa recognition from biological documents. After that, this training dataset was used to create a global standard for taxa recognition in the German biodiversity literature. Even though BIOfid represents a global standard, it is a limited resource for the following reasons: (i) input resources are limited to German literature only, (ii) the entity identification process focuses only on taxa and other more generic categories, such as person and location.

COPIOUS (Nguyen et al. 2019) is another gold standard corpus covering a wide range of biodiversity entities. The corpus has 668 documents downloaded from the Biodiversity Heritage Library with over 26K sentences and more than 28K entities. Only two annotators manually annotated the corpus with five categories of entities, i.e. taxon names, geographical locations, habitats, temporal expressions and person names. The proposed gold standard supported the development of named entity recognition and relation extraction using two different machine-learning techniques.

Species-800 (Pafilis et al. 2013) is based on 800 PubMed abstracts, such as 100 abstracts from journals in eight categories: bacteriology, botany, entomology, medicine, mycology, protistology, virology, and zoology. Similar to (Nguyen et al. 2019), Species-800 is annotated with taxon entities and normalised to the NCBI Taxonomy database.

Linnaeus (Gerner et al. 2010) is a 100 full-text documents from the PubMed Central Open Access (PMC OA) document set randomly selected and annotated for species mentions. The corpus was only annotated for species (except for the cases where genus names were incorrectly used when referring to species). Same as the case with COPIOUS and Species-800, all mentions of species terms were manually annotated and normalised to the NCBI taxonomy IDs of the intended species, except for terms where the author did not refer to the species.

QEMP (Löffler et al. 2020) is the only corpus that is based on biodiversity metadata files. It provides annotations for four main categories: Organism, Material for chemical compounds, Process for chemical, biological and natural processes, Environment that represents the habitat of organisms, Quality for data measures and Location.

The existing datasets have several limitations. They focus on species only, like the case of BioFid and COPIOUS. They are based on legacy data, as in COPIOUS and BioFID. They rely on only Pubmed abstracts like the case of Species800 and Linnaeus. They miss one important concept in the field, like the case of QEMP; it does not contain species. In this work, we create an NER corpus that contains various biodiversity classes for abstracts and metadata files.

**Relation Extraction (RE) Corpora**

Identifying the important entities is the first step in creating an RE gold standard. Based on this information, relationships amongst the entities in a sentence can be determined in a second step. There is a variety of approaches in the biomedical domain to identify relations amongst genes, diseases, proteins and drugs BioInfer (Pyysalo et al. 2007), BioRelEx (Khachatrian et al. 2019), EU-ADR (van Mulligen et al. 2012) and its successor GAD (Bravo et al. 2015). All of them use biomedical abstracts or full articles from PubMed as data sources. In contrast, some approaches do not identify the exact mention of relation but only determine the existence of a binary relation between entities (van Mulligen et al. 2012, Bravo et al. 2015, Khachatrian et al. 2019). Other gold standards distinguish between four main relation types, such as "causal", "is_a", "part_of" and "observation" (Pyysalo et al. 2007). They also developed a large ontology to describe the entities and their relations semantically.

There are only two approaches for relation extraction in the biodiversity domain: BacteriaBiotop (Delėger et al. 2016) and COPIOUS (Nguyen et al. 2019). The former defines a binary "lives_in" relation between Taxons and Habitats. The latter uses a pattern-based system that can identify any binary relations between entities within a single sentence to detect four relations: Taxon "occur" Habitat, Taxon "occur" Temporal Expression, Taxon "occur" Geographic Location and Taxon "seen by" Person.

To the best of our knowledge, there is no gold standard with relations also from dataset metadata. The introduced corpora only have the main focus on species, habitats and locations. However, biodiversity research is a diverse research field with other important categories, such as data parameters, processes, materials and data types (Löffler et al. 2021). Therefore, we aim to develop a gold standard that supports both multiple and binary relations and goes beyond the annotation of species, habitats, and geographic locations.

## General description

### Purpose

This project aims at constructing two corpora for NER and RE tasks, based on abstracts and metadata files from Biodiversity datasets.

### Additional information

**Methodology**

In this section, we describe the process of constructing the NER and RE corpora.

**BiodivNER Construction Pipeline**

**Figure 3.**
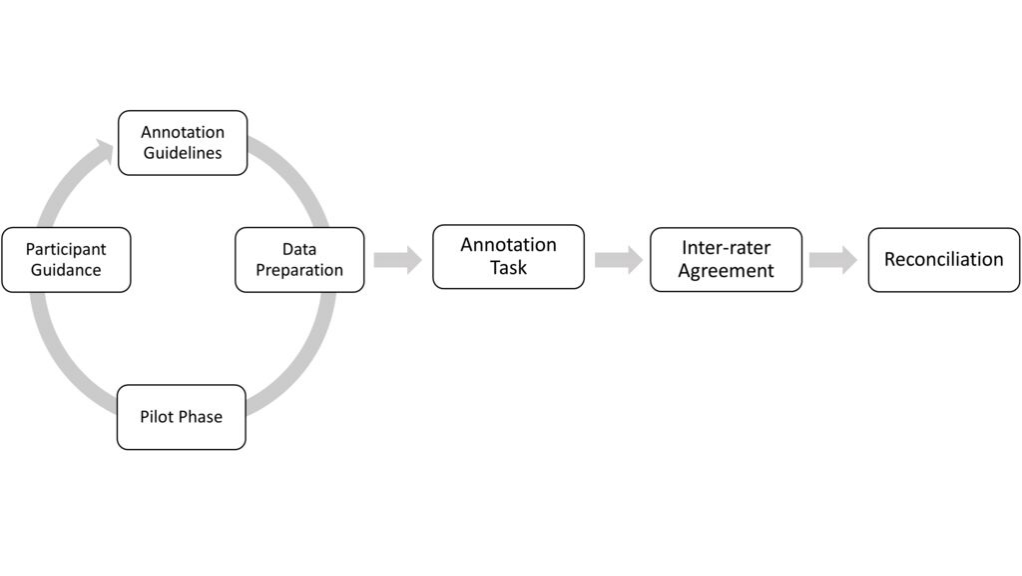
Our proposed NER corpus construction pipeline following (Pustejovsky and Stubbs 2012).

In this section, we explain the construction pipeline of the NER corpus as shown in Fig. 3. Our process consists of seven steps. It starts with the annotation guidelines to describe what we annotate and is followed by the data preparation step in which the originally collected data is transformed into the required data format used for annotation. In the pilot phase, we carry out an initial annotation task to check whether we have to modify the annotation guidelines or whether we have to invest more time in the annotators' training. Afterwards, the actual annotation task takes place. The outcome is evaluated with the computation of the inter-rater agreement. Finally, we discuss the mismatches with biodiversity experts in the reconciliation phase.

- Annotation Guidelines

We followed a modified version of our previous project guidelines to construct the QEMP corpus (Löffler et al. 2020). We set the current sentence as the only available context to annotate. We did not consider the entire document as in the gold standard construction process in NLP. Since the main purpose of this work is to develop a corpus for NER, we consider only noun entities and discard adjective entities. In addition, we gave higher attention to the complex words and minimised the chance of having two valid annotations for one term. Thus, we followed the longest span annotation and avoided nested entities annotation. For example, "benthic oxygen uptake rate" is annotated as [QUALITY], while we ignored any simple word annotation inside such span. Conjunctions are handled as two separate entities. For example, "(phylogenetic diversity)[QUALITY] of (bacteria)[ORGANISM]". We included more existing external resources than the ones used in QEMP to find proper annotations. For example, we considered the following ontologies that were used for constructing the original version of BiodivOnto: ECSO and ECOCORE for environmental-related terms, BCO and CBO for phenomena-related keywords. In addition, we utilise NCBITaxon and FLOPO for species and phenotype annotation, respectively. Moreover, we used the SWEET ontology to capture any missing terms from the previous ontologies. Our last option to find annotations from existing sources is a reference to the ontological issues detected and summarised by (Löffler et al. 2020). Such a mixture of selected resources facilitated the detection of a wide range of terms that vary in their granularity (too specific vs. too general terms).

- Data Preparation

**Figure 4.**
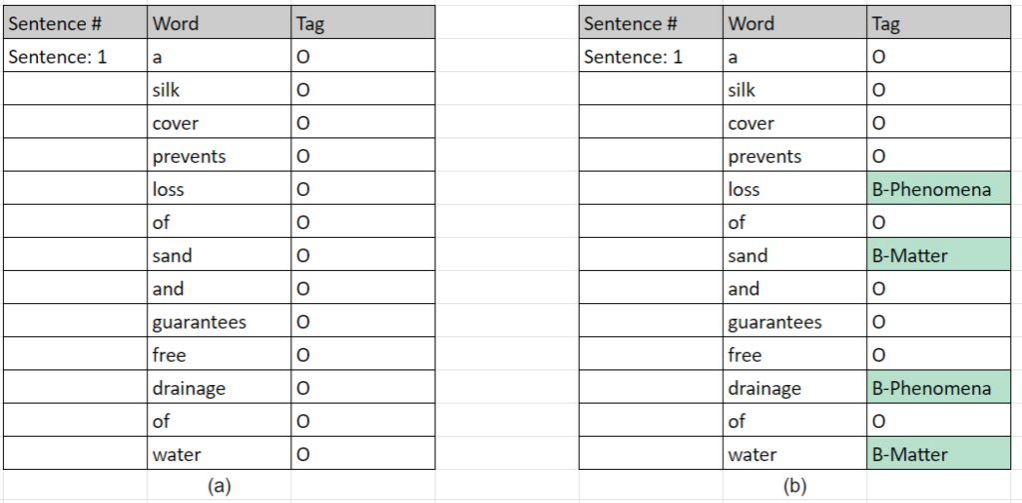
NER annotation process **a** initially prepared data; **b** while annotating data.

We parsed the original data collection into sentences. For each sentence, we tokenised it into a set of words using ntlk library. Since our used annotation format is BIO-scheme, where a word is annotated either with *B-tag* as a beginning of an entity or, *I-tag* as an inside of entity or, *O* as outside of the entity, each word is initialised with an O tag. Each sentence as a set of words with O tags is stored vertically in a CSV file, as shown in Fig. 4a. Afterwards, we split the entire corpus into two halves to enable the double annotation process.

- Pilot Phase and Participant Guidance

Four authors of this paper were responsible for annotating the corpus. Two authors have previous experience with biodiversity text annotation. The four annotators received periodical guidance from two biodiversity experts. Initially, we established a trial or a pilot phase before the actual annotation process took place. The purpose of this phase is to ensure the training of the annotators (participant guidance) as well as to revise the annotation guidelines. Around 2% (450 sentences) of the entire corpus is assigned to each annotator pair. Each annotator labelled a local copy of the pilot phase data in an Excel file. During this process, each annotator was asked to annotate a relevant term with one and only one tag from the provided tags. The results of this process are represented in Fig. 4b. After the end of the Pilot Phase, we held a "Share Thoughts" meeting to discuss the outcome. At this stage, we realised that we need a modified version of the guidelines. For example, at the beginning, not all annotators followed the 'longest span' rule and annotated every single word separately. Thus, we have settled on the longest span sequence to avoid or minimise such inconsistencies. In addition, we have decided to add the SWEET ontology to include missing terms from the other used ontologies.

- Annotation Process and Agreement

**Figure 5.**
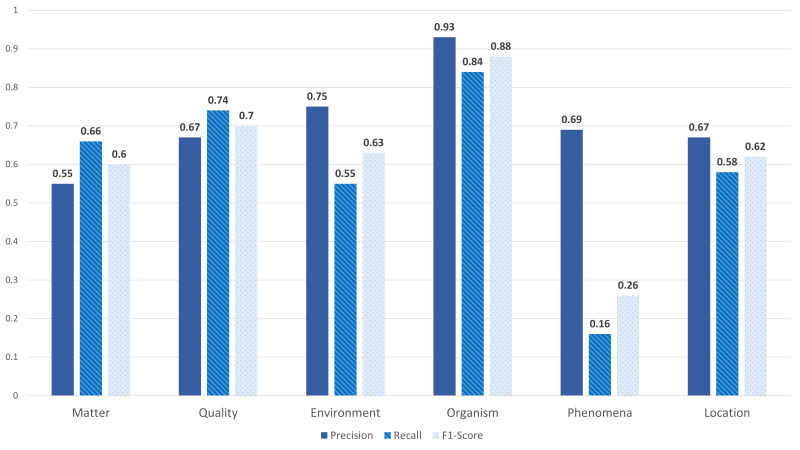
**Team A** Agreement Scores

**Figure 6.**
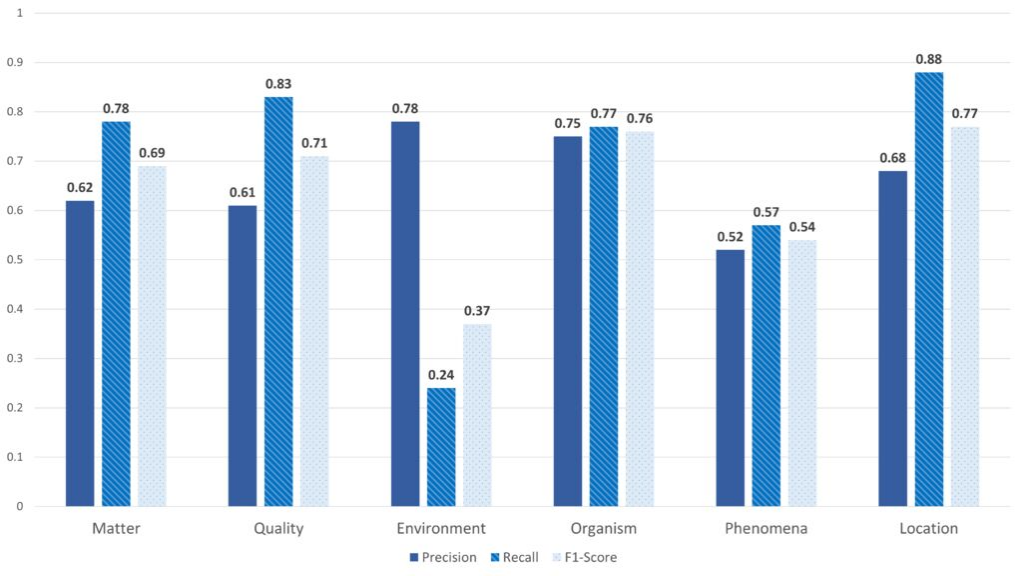
**Team B** Agreement Scores.

After the pilot phase, we familiarised ourselves with the annotation process and the guidelines. Each half of the corpus was assigned to an annotator pair. We followed the same procedure as in the pilot phase. Each annotator from the annotators' pair worked blindly on a local copy of the sheet. We refer to blindly as without access to the annotation of the other colleague. This procedure ensures the higher quality of annotated data and allows the calculation of the inter-rater agreement. Each annotator was asked to complete the annotation of half of the corpus. This annotation process was time-consuming and lasted for several months. Annotating a term is considered to be done if the annotator found the target tag in the selected existing data sources. However, if the annotator was unsure about the correct annotation, the term with a suggested tag was kept in a separate sheet named "Open Issues". We held various meetings with the biodiversity experts during this stage to solve the open issues. Since we had two annotator pairs, let's say, team A and B for two different sheets, where each sheet represented half of the corpus, we were able to calculate the inter-rater agreement for each team. We used Kappa's score for the agreement computation since it is one of the most common statistics to test inter-rater reliability (Berry and Mielke 2016). The scores are 0.76 and 0.70 for teams A and B, respectively, with an average score of 0.73. In addition, we calculated both precision, recall and F1-score for both teams, as shown in Figs 5, 6. Team A reached an average precision, recall and F1-score of 0.73, 0.65 and 0.67 respectively. However, Team B gained average scores: 0.66, 0.74 and 0.67 for both precision, recall and F1-score respectively.

- Reconciliation

We have extracted the mismatches in a separate sheet per annotator pair. A sheet contained the actual sentence with each of the annotator's answers. The task of each annotator pair was to reconcile their mismatches and to reach a final annotation that the two agreed on. We noticed that a significant cause for the mismatches was the rule of longest text span consideration in the annotation guidelines. For example, one annotator labelled the entire phrase "Secondary Metabolites" as MATERIAL, while the other tagged only "Metabolites" as MATERIAL. Such cases were the easiest to solve. However, other cases, where an annotator pair could not agree on one correct annotation were discussed with the biodiversity experts. For example, "Soil lipid biomass" seemed to be confusing as it could be either classified as MATTER or QUALITY. In such a case, we followed the biodiversity expert's opinion and settled on MATTER.

**BiodivRE construction Pipeline**

In this section, we describe our pipeline of constructing the binary and multi-class RE corpus on top of the BiodivNER. Initially, we transformed the annotated data for NER to suit the RE annotations process. Then, we tried to sample a subset of sentences to obtain a reasonable size of the RE corpus to be annotated. For each sampling method, we detailed its advantages and disadvantages. Afterwards, we explained the annotation process for the RE corpus.

- Initial Construction

**Figure 7.**
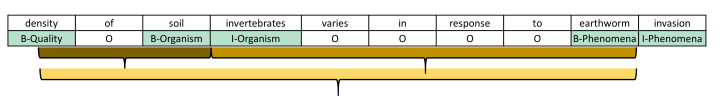
Creating sentence variations from a sentence containing more than two tags.

We considered the final NER corpus as an input for the RE corpus construction. We prepared the data in such a way to be more readable. Each sentence is represented by one row, followed by its corresponding NER annotations in the following line. The NER corpus contains sentences with multiple tags. However, an RE corpus should be designed in a way that each sentence contains exactly two tags. We generated all possible combinations for sentences with more than two tags, including exactly two tags. Fig. 7 illustrates an example where one sentence with three tags generates three sentences with two labels. This operation generated a large-scale corpus with more than 52K sentences. We expect a high rate of FALSE (no relation) statements in the generated corpus. However, our task aims at creating an RE corpus with a good balance between TRUE (existing relation) and FALSE sentences. To achieve this, we have to choose a suitable sampling strategy to achieve the best balance amongst the selected sentences. Therefore, we have explored two different sampling methods. We discuss them in the following sections.

- Random Sampling

In the pilot phase of BiodivRE construction, we used a random sampling mechanism amongst the created corpus. We did not consider any selection criteria. We directly stacked the entire corpus in a list, shuffled it and randomly picked "n" sentences. We started annotating the resultant smaller corpus and, by doing so, we encountered two issues. At first, we found long sentences with too far tags, i.e. have many words between them, which makes the existence of a relation between the two tags impossible. Second, some of the relation pairs in the ontology have not appeared in the corpus at all. There are two reasons for the second issue. Either such kinds of relations do not appear in the original corpus or they are missed by the sampler since it depends purely on the random selection. The conclusion from the pilot phase is the need for changing the sampling strategy.

- Balance-Biased Sampling

We developed a Balance-Biased sampler to have more control over what to include in the final RE corpus. It is inspired by the Round-robin scheduler. We grouped the sentences from the initial construction by tag-pair, where a valid pair is the one appearing in the BiodivOnto and the unsupported co-occurrences were grouped into a new category, "Other". At this stage, we handled the relations bidirectionally between entities of interest to cover cases like ENVIRONMENT have QUALITY and QUALITY of ENVIRONMENT. Afterwards, we iterated over the groups, including the entire set of tag-pairs, as well as the "Other" group. We picked one sentence from each group until a threshold was reached. By this means, we avoided any bias that could be caused by a random sampler. In our case, we selected 4000 sentences as a threshold. An additional criterion is that we limit the number of words between the two entities of interest to a certain value, for example, 30 words. In this way, we solved the two problems that appeared using the random sampling method. At first, we guarantee that we cover all the relations of the BiodivOnto, if it exists in the text, in the final corpus. Second, we avoid cases with FALSE sentences due to too far entities, since it is clear that no relation could exist between them.

- Annotation Process

We directly referred to BiodivOnto and limited the accepted relations to those supported by the ontology. On the one hand, for each sentence, we checked whether there is a relation between its two named entities. On the other hand, whether this relation has a semantic correspondence in the BiodivOnto. For example, a verb relation "has an impact on" is considered a synonym for the ontological relation "influence". FALSE examples would be either the relation is not supported by the BiodivOnto or it has a different meaning than the ontological relation. For example, "Climate change (B-Phenomena I-Phenomena) impacts the carbon dioxide (B-Matter I-Matter)" is a FALSE sentence since there is no ontological relation between PHENOMENA-MATTER. Such a sentence would appear since we also choose from the "Other" group in the selected sampling method. Another FALSE example might occur between two entities with a relation in the BiodivOnto. "Trees (B-Organism) with extrafloral nectaries (B-Matter I-Matter)" is a FALSE statement since the word with does not imply the relation influence between ORGANISM and MATTER.

Similar to our procedure to construct the NER corpus, we also applied a pilot phase for RE annotation. Two of the authors annotated the same 50 sentences that were randomly picked. Afterwards, we calculated the inter-rater agreement (Kappa's score), which resulted in 0.94. Due to this high score, we decided to split the corpus and individually continue the annotation.

**Figure 8.**
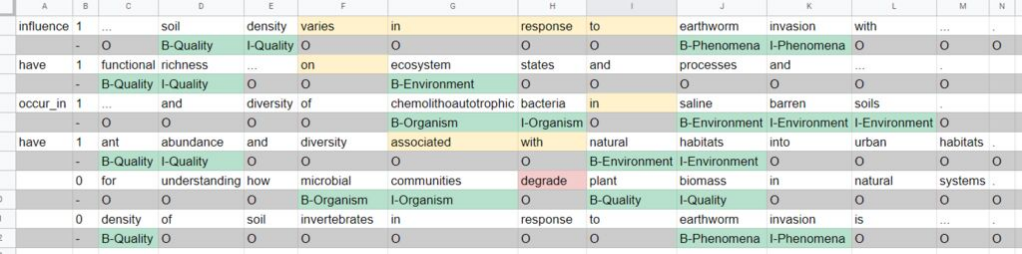
A snippet of an RE sheet during annotation.

During the real annotation phase, we encountered issues regarding the entity tags, especially for the longest span annotation. This rule does not seem to be correctly followed during the annotation of the NER corpus. For example, "earthworm invasion" was annotated as "B-Organism" "B-Phenomena", instead of "B-Phenomena" "I-Phenomena". For those cases, we fixed them to follow the rule of the annotation declared originally in the NER guidelines. Fig. 8 shows samples from an annotation sheet. The first column holds the actual relation label from BiodivOnto that will be used for the multi-class RE corpus. Then, it is followed by a binary relation tag (0- no relation, 1- existing relation). Yellow cells highlight the relation between the two entities of interest in the text. Red cell indicates that there is a relation based on the sentence, but not supported by BiodivOnto. In this sentence, the verb "degrade" has an "influence" meaning implicitly. However, we expect to have a relationship that semantically means "have"; thus, the sentence is tagged with a "0". Other sentences, like the last one, indicate no relation at all.

## Geographic coverage

### Description

Not Applicable

## Usage licence

### Usage licence

Creative Commons Public Domain Waiver (CC-Zero)

## Data resources

### Data package title

BiodivNERE

### Resource link

https://doi.org/10.5281/zenodo.6575865

### Number of data sets

3

### Data set 1.

#### Data set name

BiodivNER

#### Data format

CSV

#### Download URL

https://zenodo.org/record/6575865/files/BiodivNER.zip?download=1

#### Description

Three files per named entity recognition (NER) represent train, dev and test splits.

**Data set 1.** 

Column label	Column description
Sentence#	Number of sentence in increasing order.
Word	Tokenised sentence into words.
Tag	Corresponding NER tag that follows BIO-schema. Possible values are B/I-Environment, B/I-Phenomena, B/I-Matter, B/1-Quality and B/I-Location, B/I-Organism.

### Data set 2.

#### Data set name

BiodivRE

#### Data format

CSV

#### Download URL

https://zenodo.org/record/6575865/files/BiodivRE.zip?download=1

#### Description

Three files for Relation Extraction (RE) represent train, dev and test splits.

**Data set 2.** 

Column label	Column description
Not Applicable	Possible values are 1 for relation exisits and 0 for relation does not exist.
Not Applicable	the actual sentence with two anonymised entities that are supposed to have (have not) a relation.

### Data set 3.

#### Data set name

BiodivRE_MultiClass

#### Data format

CSV

#### Download URL

https://zenodo.org/record/6575865/files/BiodivRE_MultiClass.zip?download=1

**Data set 3.** 

Column label	Column description
Not Applicable	Possible values NA (Not Applicable where the relation is undetermined), influence, have and occur_in.
Not Applicable	the actual sentence with two anonymised entities that are supposed to have (have not) a relation.

## Additional information

**Results and Discussion**

In this section, we give an overview of our final NER and RE corpora. We illustrate the characteristics of each corpus, for example, the class distribution in the NER corpus. In addition, we compare them to existing state-of-the-art corpora.

**BiodivNER Characteristics**

**Figure 9.**
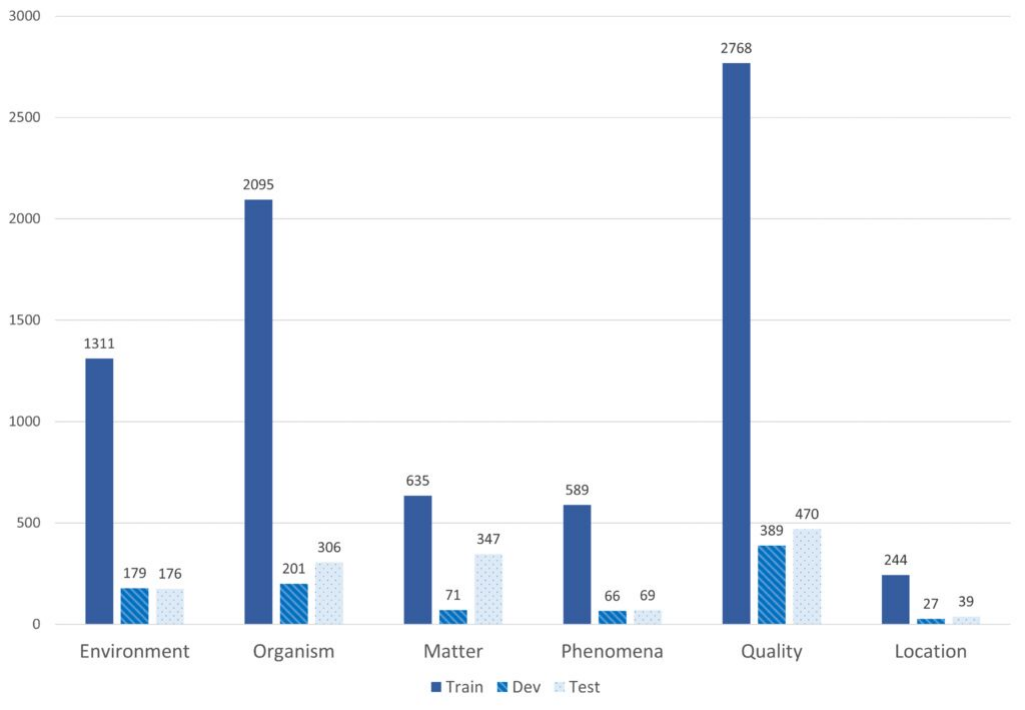
Category distribution of BiodivNER corpus.

The final version of the NER corpus consists of three folds: train, dev and test because our corpus mainly addresses various tasks in NLP that could be solved, based on machine-learning techniques. We followed the split of 80%, 10% and 10% for the train, dev and test sets, respectively. All files are given in a CSV format, each of which consists of three entries Sentence#, Word and Tag, as shown in Fig. 4b. Fig. 9 provides an overview of the category distribution inside the BiodivNER corpus in the tree data folds. QUALITY represents the most occurring mention in the corpus, followed by ORGANISM and ENVIRONMENT, respectively. However, LOCATION is the least frequent one. The overall distribution reflects a diverse corpus of the most important classes in the biodiversity domain.

Moreover, we compare our BiodivNER to the existing common corpora. Table 2 shows the comparison overview. We compared in terms of the used data source, collected data type, number of annotated documents, number of statements, words, categories and mentions. Mentions represent how many words are annotated. We also provide the number of unique mentions. COPIOUS corpus is the largest in terms of all aspects, except the number of categories. However, BiodivNER covers the greatest number of categories. In addition, BiodivNER is the largest corpus that is based on metadata files of biodiversity datasets as a data source.

COPIOUS has two categories closely related to biodiversity (Habitat and Taxon) and two general Categories (TemporalExpression and GeographicalLocation). QEMP has four categories derived from the biodiversity domain (Environment, Material, Process and Quality). As there is already a variety of corpora for species, we only concentrated on missing categories in QEMP. BiodivNER also covers such an essential category in addition to the same closely-related classes as QEMP and a general domain LOCATION category.

**BiodivRE Characteristics**

Similar to BiodivNER, we created three folds in a CSV format for both binary and multi-class RE corpus. The files consist of two columns: (1) the relationship either in a binary or label form and (2) the sentence where the actual named entities are encoded with their tags. An example line in the file of binary relations: "1 Our study shows a significant decline of the @QUALITY$ of @ENVIRONMENT$.". However, it would be in the multi-relations files as: "have, Our study shows a significant decline of the @QUALITY$ of @ENVIRONMENT$." This format will facilitate the training procedure for any machine-learning technique. We followed the same split setting for 80%, 10%, 10% of the train, dev and test sets, respectively.

**Figure 10.**
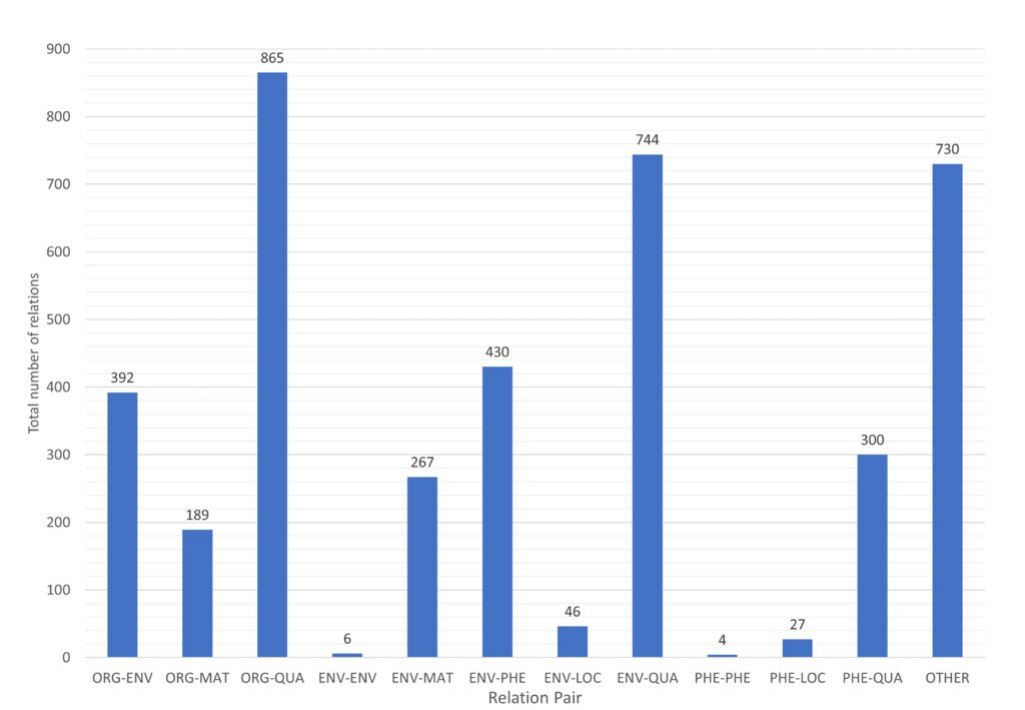
Category pairs distribution. For display purposes, category names are abbreviated to three letters.

**Figure 11.**
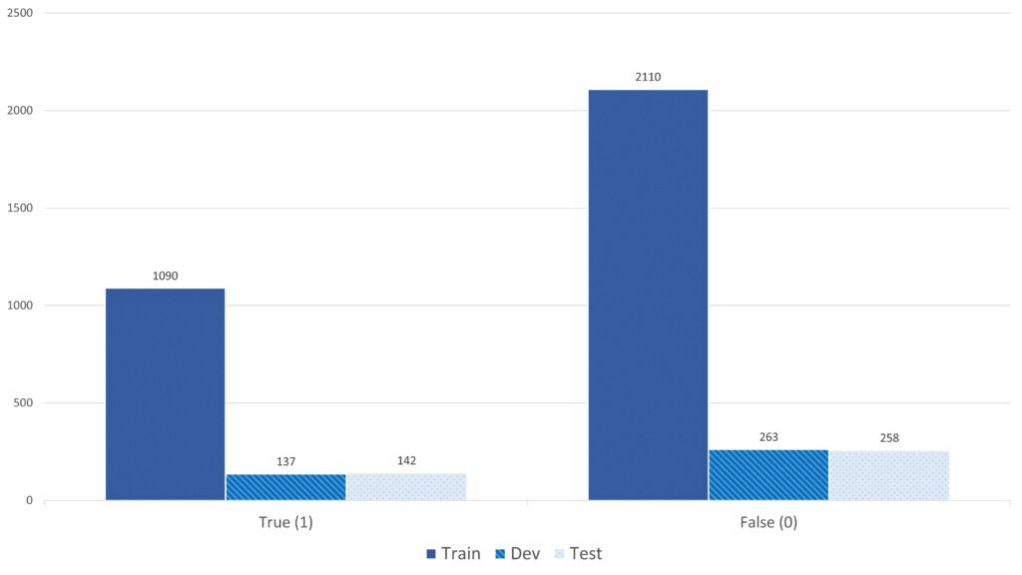
The binary distribution of the BiodivRE corpus

**Figure 12.**
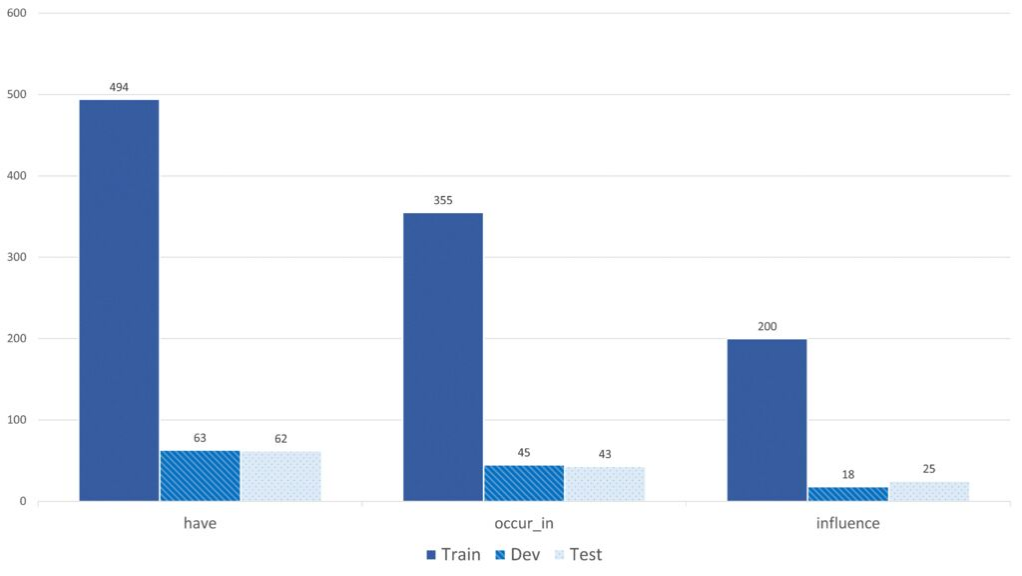
Multi-class relations distribution of BiodivRE corpus.

Fig. 10 shows the category pairs distribution of the BiodivRE corpus. We have calculated the frequencies in a bidirectional order. For example, ORG-ENV represents the total of such a pair and ENV-ORG as well. Since QUALITY is the most frequent class in the NER corpus, this is also reflected in the category pairs ORG-QUA and ENV-QUA. The self-relations that appear in ENV-ENV and PHE-PHE are the least frequent in our corpus. Other category pairs that the BiodivOnto support do not appear in the text used for creating the RE corpus. For example, ORG-ORG and ORG-LOC. The "Other" group represents any co-occurrences that appear in the text and do not exist in the BiodivOnto. In addition, Figs 11, 12 depict the binary and multi-class annotation distribution of the BiodivRE in the three folds of the benchmark. Such that "have" followed by "occur_in" are the most common relations in the corpus.

Table 3 identifies our RE corpus and the biomedical corpora GAD, EU-ADR and BioRelEx. We selected these corpora for comparison since the data are publicly available and the scope of the annotation is limited to only one sentence, as was the case of our BiodivRE corpus. For example, the COPIOUS corpus discusses the RE part, but the data are unavailable. In addition, BioCreative V (Rinaldi et al. 2016) uses the entire abstract as a context of annotation and, thus, we skip it here. For BioRelEx, in the original dataset paper, they have -1, 1 and 0 classes. We use them here as the former two classes map to TRUE, while the latter maps to FALSE classes. BiodivRE has a second-place amongst the existing corpora concerning the number of sentences (4K) with a higher rate of FALSE sentences. There are two reasons behind this high number of FALSE statements. On the one hand, we found that most metadata sentences have a listing format of entities and we could not guess the relation amongst them (the most frequent sentences). On the other hand, BiodivOnto is still incomplete; some relations are missing from it. For example, "Trees (B-ORGANISM) with extrafloral nectaries (B_MATTER, I-MATTER)" holds a meaning of contains, but we look for influence.

**Conclusions and Future Work**

We introduced BiodivNERE as a package for two corpora for NER and RE tasks that are based on abstracts and metadata from the biodiversity domain. We manually annotated and revised them with biodiversity experts. BiodivNER, the NER corpus, consists of six important classes in the biodiversity domain. BiodivRE is a binary and multi-class benchmark containing three relations from the domain. Both classes and relations are derived from the analysis of our previously-developed work (Biodiversity Questions and BiodivOnto). We release our corpora and code as publicly available.

Future Work

We see multiple areas to extend this work. We plan to include more classes and relations from the biodiversity domain. For example, we restore the dropped relations from BiodivOnto, for example, "part_of" and "is_a". In addition, we include more data sources to cover a broader range of the domain. Moreover, we evaluate them in terms of the quality of the annotations. Last but not least, we apply both corpora to a machine-learning model to bring them to the actual use case.

## Figures and Tables

**Table 1.** Summary of the categories (entity types) used for NER annotation. Explanations are adapted from (Löffler et al. 2021).

Tag	**Explanations**	**Examples**
ORGANISM	all individual life forms such as microorganisms, plants, animals	mammal, insect, fungi, bacteria
PHENOMENA	occurring natural, biological, physical or chemical processes including events	decomposition, colonisation, climate change, deforestation
MATTER	chemical and biological compounds, and natural elements	carbon, H_2_O, sediment, sand
ENVIRONMENT	Natural or man-made environments ORGANISM live in	groundwater, garden, aquarium, mountain
QUALITY	data parameters measured or observed, phenotypes and traits	volume, age, structure, morphology
LOCATION	geographic location (no coordinates)	China, United States

**Table 2.** State-of-the-art comparison of NER corpora. The number of documents, statements and categories are given by #Doc., #Stat. and #Cate. respectively.

Corpus	Data Source	Type	#Doc.	#Stat.	#Words (#Tokens)	#Cate	#Mentions (#Annotations)	#Unique Mentions
COPIOUS	BHL	Publications	668	26,277	502,507	5	26,007	6,753
QEMP	idiv, BEXIS, Pangeya, Dryad, BFChina	Dataset Metadata	50	2,226	90,344	4	5,154	480
Species-800	PubMed	Abstracts	800	14,756	381,259	1	5,330	1,441
Linneaus	PubMed Central (PMC)	Publications	100	34,310	828,278	1	3,884	324
**BiodivNER**	iDiv, BExIS, Pangeya, Dryad, BEF-china, PubMed	Dataset Metadata, Abstracts	150	2,398	102,113	6	9,982	1,033

**Table 3.** RE corpora comparison.

Corpus	Relations	#TRUE Statements	#FALSE Statements	Total
GAD	Binary	25,209	22,761	53,300
EU-ADR	Binary	2,358	837	3,550
BioRelEx	Multi-class	1,379	62	1,606
**BiodivRE**	Binary, Multi-class	1,369	2,631	4,000
